# Interactions between NLRP3 inflammasome and glycolysis in macrophages: New insights into chronic inflammation pathogenesis

**DOI:** 10.1002/iid3.581

**Published:** 2021-12-13

**Authors:** Qun Yu, Maojuan Guo, Wenyun Zeng, Miao Zeng, Xiaolu Zhang, Yue Zhang, Wenlan Zhang, Xijuan Jiang, Bin Yu

**Affiliations:** ^1^ School of Integrative Medicine Tianjin University of Traditional Chinese Medicine Tianjin China

**Keywords:** glycolysis, inflammation, macrophages, NLRP3 inflammasome

## Abstract

NLRP3 inflammasome activation in macrophages fuels sterile inflammation, which has been tied with metabolic reprogramming characterized by high glycolysis and low oxidative phosphorylation. The key enzymes in glycolysis and glycolysis‐related products can regulate and activate NLRP3 inflammasome. In turn, NLRP3 inflammasome is considered to affect glycolysis, as well. However, the exact mechanism remains ambiguous. On the basis of these findings, the focus of this review is mainly on the developments in our understanding of interaction between NLRP3 inflammasome activation and glycolysis in macrophages, and small molecule compounds that influence the activation of NLRP3 inflammasomes by regulating glycolysis in macrophages. The application of this interaction in the treatment of diseases is also discussed. This paper may yield valuable clues for development of novel therapeutic agent for NLRP3 inflammasome‐related diseases.

## Graphical abstract

The activation of NLRP3 inflammasome in macrophages is crucial in inflammation. The key enzymes in glycolysis and glycolysis‐related products can regulate and activate NLRP3 inflammasome. The NLRP3 inflammasome plays a regulatory role in glycolysis, as well.

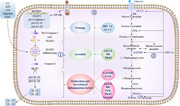

## 1 INTRODUCTION

Macrophages play a major role in some chronic inflammatory conditions, such as atherosclerosis,^1^ aging,^2^ type 2 diabetes,^3^ rheumatoid arthritis,^4^, ^5^, ^6^ and obesity.^7^ Innate immunity is well known as the first line of host defense against pathogens. Upon encountering antigen, pattern recognition receptors (PRRs) are responsible for recognition of pathogen‐associated molecular patterns (PAMPs)/damage‐associated molecular patterns (DAMPs) and then trigger proinflammatory effector.^8^ The NOD‐like receptors (NLRs) are critical cytoplasmic PRRs that also functions in the host innate immune response. NLRs including NLRP1, NLRP3, NLRC4, and absent in melanoma 2 (AIM2) belong to critical cytoplasmic PRRs, which act in the form of inflammasomes.^9^, ^10^ Among all inflammasomes, the best characteristic one is NLRP3 inflammasome.^8^ Activation of the NLRP3 inflammasome and its downstream pathway to reduce pro‐caspase‐1 activation and caspase‐1‐mediated interleukine‐1β (IL‐1β) maturation in macrophages plays a contributing role in the progression of inflammation.^11^, ^12^, ^13^

In the past few years, metabolic reprogramming attracts widespread attention among immunologist.^14^ Recently, the relationship between inflammatory profile and the metabolic perturbations in glucose metabolism has attracted more attention.^15^ Under inflammatory triggers, macrophages undergo metabolic shift from oxidative phosphorylation to glycolytic metabolism,^16^, ^17^, ^18^ which implies that glycolysis exerts a negligible effect in NLRP3 inflammasome activation in macrophages.^8^ The activated NLRP3 inflammasome in turn affects key enzymes of glycolysis,^19^ thereby regulating glycolysis flux.^20^ Here, we elaborate the interactions between NLRP3 inflammasome activation and glycolysis in macrophages, which is helpful toward a better understanding of complex mechanisms of inflammasome activation and finding the potential therapeutic benefits of targeting them in chronic inflammatory diseases.

## 2 AN OVERVIEW OF NLRP3 INFLAMMASOME AND GLYCOLYSIS

NLRP3 inflammasome, a multiprotein cytoplasmic complex, include NLRP3, apoptosis‐associated speck‐like protein (ASC), and pro‐caspase‐1.^21^ NLRP3, as the core component of NLRP3 inflammasome, contains leucine‐rich repeat sequence responsible for stimulation recognition; nucleotide‐binding oligomerization domains (NODs) that drive self‐oligomerization; and pyrin domain (PYD) that acts as intermediary in interactions between NLRP3 and ASC proteins through homotypic PYD‐PYD domains. Recent results in inflammation research have identified the critical role of NLRP3 inflammasome in macrophages responding to chronic inflammation.^22^, ^23^ NLRP3 specific ligands such as cholesterol crystals (CC), ATP, Lipopolysaccharidepore (LPS), forming toxins (nigericin) and monosodium urate (MSU) crystals, activate NLRP3 inflammasome.^21^, ^24^, ^25^ It is often assumed that NLRP3 inflammasome activation in macrophage requires two steps: “priming” and “activation.” The priming phase induces pro‐IL‐1β synthesis and NLRP3 upregulation.,^26^ while during the activation phase the oligomeric NLRP3 inflammasome complex assembles, thereby cleaving IL‐1β and IL‐18. IL‐1β and IL‐18 trigger inflammatory cascades, and so amplify the inflammatory reaction.^27^

Glycolysis, as a universal biochemical process, converts glucose into pyruvate and produces two ATPs.^28^, ^29^ Lactate dehydrogenase A (LDHA) catalyzes the reaction that converts a portion of pyruvate into lactate.^30^ Among all reactions of glycolysis, three rate‐limiting reactions are catalyzed by hexokinase (HK), phosphofructokinase (PFK), and pyruvate kinase (PK). The expression of glucose transporter (GLUT) determines the rate of glucose uptake. The most abundant and ubiquitous GLUT in macrophages is GLUT1,^31^ which is rapidly upregulated in inflammatory microenvironment to participate in its switch to glycolytic phenotype.^32^ Then, HK1 isoform, ubiquitously expressed in most tissues,^33^ phosphorylated glucose to glucose‐6‐phosphate in cytoplasm, the first and rate‐limiting step in glycolysis.^34^ PFK1 catalyzes the second speed‐limiting step of glycolysis that phosphorylated fructose‐6‐phosphate to fructose‐1, 6‐bisphosphate.^15^ PFK‐1 is allostericly activated by fructose‐2,6‐bisphosphate (F2,6BP) that is controlled by 6‐phosphofructo‐2‐kinase /fructose‐2,6‐bisphosphatase 3 (PFKFB3) to balance its amount.^35^ As an evolutionary conserved metabolic enzyme, PK catalyzes the production of pyruvate from phosphoenolpyruvate.^36^ Mammalian PK contains four isoforms: red blood cell PK (PKR), liver‐type PK (PKL), and PK muscle isozyme M1 and M2 (PKM1 and PKM2).^37^ PKM2 is mainly abundant in hyper‐proliferative cells such as the majority of tumor cells and macrophages.^38^ Besides HK, PFK and PK, recent metabolite flux analyses revealed that glyceraldehyde‐3‐phosphate dehydrogenase (GAPDH) is also a key enzyme of glycolysis under nutrient‐rich conditions by converting glyceraldehyde 3‐phosphate to 1, 3‐biphosphoglycerate and exerts flux control over the glycolytic pathway.^39^ The dehydration of 2‐phospho‐d‐glycerate (2‐PG) to phosphoenolpyruvate (PEP) in the glycolysis pathway is catalyzed by enolase (ENO), a metalloenzyme, leading to enhanced glycolysis.^40^ Alpha‐enolase (ENO1) isoform is expressed in most tissues but exhibits non‐glycolytic functions in macrophages.^41^

## 3 INTERACTIONS BETWEEN GLYCOLYSIS AND NLRP3 IN MACROPHAGES

A switch of oxidative phosphorylation to aerobic glycolysis is a possible respond of immune cells to inflammation.^42^ It has been suggested that macrophages undergo metabolic reprogramming to maintain immunologic and defensive functions and proinflammatory macrophages up‐regulate rate of glycolysis rapidly,^43^ but its role in inflammasome activation is ambiguous.^44^ It was reported that the canonical activation of NLRP3 inflammasomes depends on glycolysis.^45^ In contrast, other studies have suggested that the inhibition of glycolytic enzymes such as GAPDH, ENO1, and HK leads to NLRP3 inflammasome activation.^20^, ^46^ Meanwhile, NLRP3 inflammasome activation also regulates a series of glycolytic enzymes thereby affecting the corresponding process of glycolysis.

### 3.1 Glycolysis may regulate the activation of macrophage NLRP3 inflammasome

**Figure 1:**
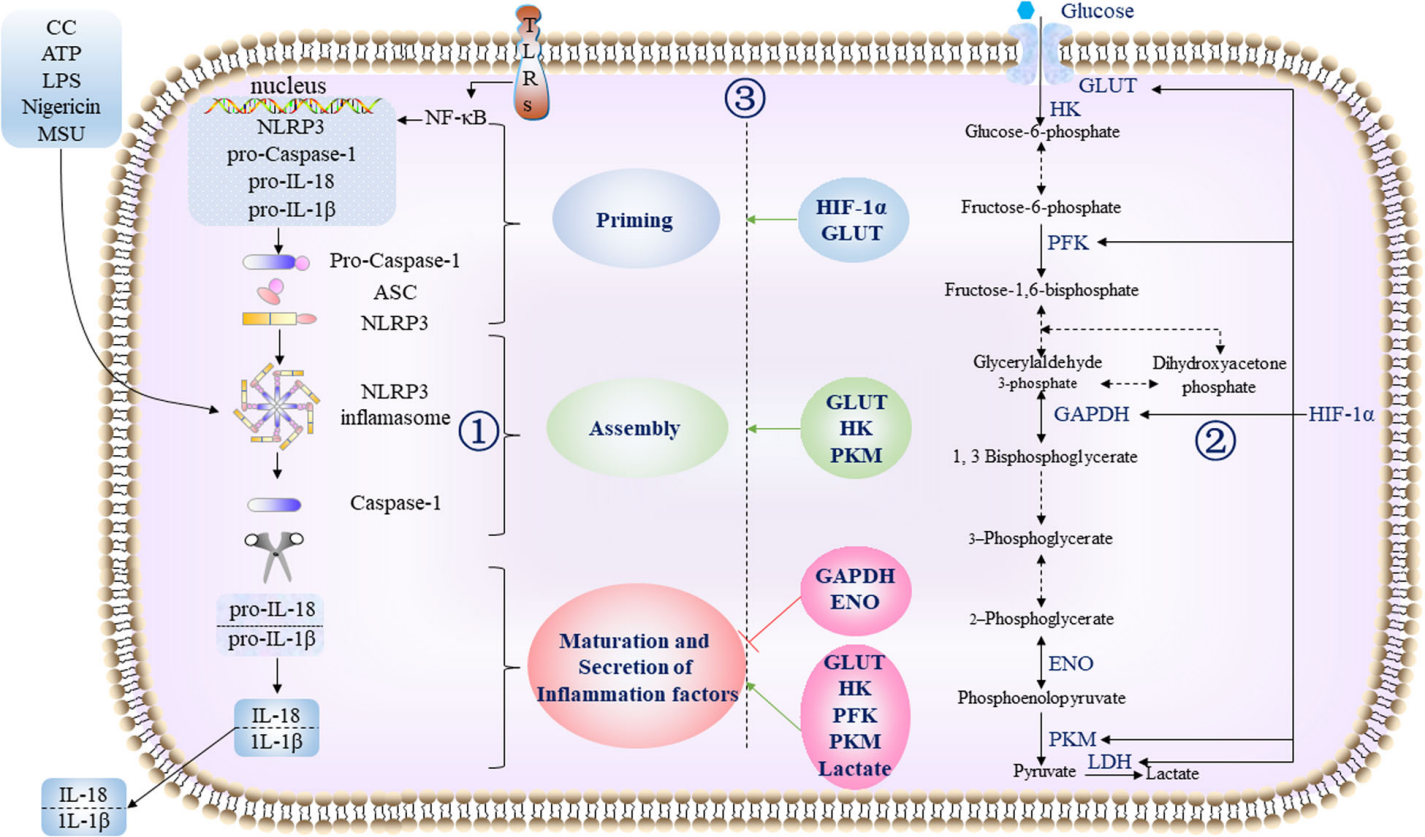
Schematic representation of the possible mechanism of glycolysis regulates NLRP3 inflammasome in macrophages. ① NLRP3 inflammasome activation is a two‐step process, with both priming and activation and NLRP3 must be primed before activation. In priming stage, an NF‐κB–activating stimulus, such as LPS binding to TLRs, induces high expression of NLRP3, pro‐Caspase‐1, pro‐IL‐1β, and pro‐IL‐18, which leads to increased expression of their proteins. After priming, canonical NLRP3 inflammasome activation requires a second, distinct signal to activate NLRP3 and lead to the formation of the NLRP3 inflammasome complex. NLRP3 specific ligands can also activate NLRP3 inflammasome. As a result, pro‐caspase‐1 is converted to Caspase‐1. Upon activation, active Caspase‐1 cleaves the pro‐IL‐1β and pro‐IL‐18 into their mature forms, which secret out of cells. ② Glycolysis is a biological process that occurs to convert glucose into pyruvate to provide energy for cells. Since the glycolysis cycle involves the conversion of blood sugar into an anion of pyruvic acid (pyruvate), glycolysis is also referred to as the citric acid cycle under hypoxia condition or aerobic glycolysis. Aerobic glycolysis refers to the process of glycolysis under aerobic conditions. When aerobic glycolysis occurs under hypoxic conditions, it called anaerobic glycolysis. There are ten reactive steps to occur that involve several catalyst enzymes such as HK, PFKKM and PKM and intermediate compounds. HIF‐1a is a key regulator of glycolysis during hypoxia, upregulate the coding of aerobic glycolysis enzyme at the transcription level in macrophages. ③ HIF‐1a, GLUT, HK, and PK induce the priming step of NLRP3 inflammasome. GLUT, HK, and PK promote NLRP3 inflammasome assembly. GLUT, HK, PFK, PK, and lactate, promote inflammatory factors secretion mediated by NLRP3 inflammasome activation. GAPDH and ENO inhibit inflammatory factors secretion mediated by NLRP3 inflammasome activation. ASC, apoptosis‐associated speck‐like protein; ENO, enolase; GAPDH, glyceraldehyde‐3‐phosphate dehydrogenase; GLUT, glucose transporter;, HIF‐1a, hypoxia inducible factor‐1α; HK, hexokinase; IL‐18, interleukine‐18; IL‐1β, interleukine‐1β; LDH, lactate dehydrogenase; LPS, lipopolysaccharide; NF‐κB, nuclear factor κβ; NLRP3, Nod‐like receptor protein 3; PFK, phosphofructokinase; PKM, pyruvate kinase muscle isozyme; TLRs, Toll‐like receptors

Glycolysis presents a major regulatory effect in the activation of NLRP3 inflammasome.^45^, ^47^ During glucose metabolism reprogramming in macrophages, the switched activation of key glycolytic enzymes is key in regulating NLRP3 inflammasome activation. However, it is still unclear whether glycolytic cascade regulates NLRP3 inflammasome activity positively or negatively.^20^, ^45^, ^48^, ^49^ Several glycolysis regulators have been involved in NLRP3 inflammasome activation (Figure 1). Previous research has established that some small molecule compounds could regulate glycolysis in macrophages and affect the activation of NLRP3 inflammasome (Table 1).

**Table 1:** Some compounds regulate glycolysis and then inhibit NLRP3 inflammasome‐dependent inflammation in macrophages

Target	Compound	Mechanism	Effect on NLRP3	References
HK	2‐DG	Promotes HK2 dissociation from the outer mitochondrial membrane	Inhibits the expression of caspase‐1, IL‐1β and IL‐18	[45]
	ATRA	Enhances HK2 expression	Reduces NLRP3 inflammasome‐dependent IL‐1β secretion	[50]
	Andrographolide	Inhibits the activity of HK2	Reduces the release of IL‐1β	[51]
PK	Shikonin	Inhibits EIF2AK2 phosphorylation	Reduces caspase‐1 activity, and IL‐1β and IL‐18 release	[47]
	LBP	Reduces the expression of PKM2 protein	Reduces IL‐1β production	[52]
	DET	Inhibits the nuclear localization of PKM2	Attenuates the release of IL‐1β	[53]
	IRD	Targets PKM2 and inhibits its downstream expression	Inhibits the release of IL‐1β	[54]
HIF‐1α	Chaetocin	Inhibits HIF‐1α expression	Suppresses priming of NLRP3 inflammasome and IL‐1β synthesis	[55]
GAPDH	GB111‐NH2 KB	Inhibits GAPDH expression, thereby decreases glycolytic flux	Inhibits inflammatory factors maturation and release	[20]
α‐enolase	GB111‐NH2 EB	Inhibits α‐enolase expression, then decreases glycolytic flux	Induces inflammatory factors maturation and release	[20]
LDHA	GSK2837808a	Effective and selective inhibitor of lactate dehydrogenase A (LDHA)	Reduces the protein levels of mature IL‐1β and active caspase‐1	[24]

Abbreviations: DET, deoxyelephantopin; GAPDH, glyceraldehyde‐3‐phosphate dehydrogenase; HIF‐1a, hypoxia inducible factor‐1α; HK, hexokinase; IL‐18, interleukine‐18; IL‐1β, interleukine‐1β; LBP, *Lycium barbarum* polysaccharide; NLRP3, Nod‐like receptor protein 3; PK, pyruvate kinase; PKM, pyruvate kinase muscle isozyme.

#### 3.1.1 GLUT

As the most abundant glucose transporter in macrophages,^31^ GLUT1 induces the full activation of NLRP3 inflammasome. Its silence by pharmacological inhibition (STF‐31: iGLUT1) or gene knockdown results in attenuated phorbol myristate acetate (PMA)‐induced gene expressions of both NLRP3 and IL‐1β, suggesting that GLUT1 involves in the priming step of NLRP3 inflammasome.^32^ Additionally, inhibition of GLUT1 reduces glucose uptake and restrains glycolysis in macrophages, prevents ASC speck formation and NLRP3 inflammasome assembly.^32^ GLUT1 knockdown also reduces maturation and release of IL‐1β in macrophages.^56^ The reduction of GLUT1 in vivo is regulated by the proto‐oncogene Casitas B‐lineage lymphoma (Cbl) through posttranscriptional modification. Cbl encodes an ubiquitin ligase, and mediates protein ubiquitination.^57^ The expression of GLUT1 is upregulated, thereby promotes maturation and secretion of IL‐1β and actives caspase‐1 in THP‐1‐derived macrophages with Cbl‐KO. In summary, the above research shows that GLUT1 helps in both of the priming and activation of the NLRP3 inflammasome.

#### 3.1.2 HK

HK, as the first speed‐limiting enzyme in the glycolytic pathway, activates NLRP3 inflammasome to promote the maturation and secretion of inflammatory factors. So far, HK isozymes, named from HK1 to HK4, have been identified in mammals.^58^ HK can be incorporated into the outer membrane of mitochondria through the interaction with the voltage‐dependent anion channel (VDAC) at, a pattern recognition receptor.^46^ Among the three VDAC identified subtypes (VDAC1‐3), VDAC1 is widely expressed in a vast many cell types.^59^ Dissociation of HK from VDAC1 induces inflammasome assembly, then the maturation and release of IL‐1β and IL‐18. HK1 knockdown inhibits caspase‐1 activation, IL‐1β and IL‐18 release stimulated by LPS/ATP in murine macrophages J774A.1.^45^ Decreased expression of HK1 mitigates caspase‐1 activation, therefore reducing secretion of IL‐1β and IL‐18 in wild type bone marrow–derived macrophages (BMDMs) in response to joint stimulation by LPS and ATP.^45^ Some agents that inhibit HK dissociating from the mitochondrial membrane or expression suppress NLRP3 inflammasome activation. As a derivative of vitamin A, all‐trans retinoic acid (ATRA) enhances HK2 expression, and converts the cellular metabolism of macrophages to glycolysis in LPS treatment, which promotes NLRP3 inflammasome activation. What's more, 3‐bromopyruvate (3BP), as selective inhibitor of HK2, attenuates IL‐1β secretion induced by ATRA in macrophages.^50^ As the major negative regulator of autophagy,^60^, ^61^ mammalian target of rapamycin complex 1 (mTORC1) induces HK1 expression via phosphorylation of 4E‐binding protein 1(4E‐BP1), the eukaryotic translation initiation factor.^45^, ^60^, ^62^ In line with this, HK1 expression can be inhibited by Torin1, the mTOR selective inhibitor^63^ or rapamycin^64^ or knockdown the 4E‐BP1 and Raptor, a scaffold for mTORC1 complex substrates.^65^ Carbon monoxide‐releasing molecules (CORM) inhibits mTORC1 activation, thereby inhibits glycolysis during NLRP3 inflammasome activation,^66^ inhibits ASC oligomerization and caspase‐1 activation, and decreases secretions of IL‐1β and IL‐18 in macrophages stimulated by LPS and ATP. Elevated levels of glucose‐6‐phosophate (G6P) also promotes HK release from mitochondria, thus slows the rate of G6P production.^67^, ^68^ Acting as a d‐glucose mimic, 2‐deoxyglucose (2‐DG) is phosphorylated by HK to 2‐deoxyglucose‐6‐phosphate (2‐DG6P), which inhibits the function of HK in a way similar to G6P. As an immune checkpoint molecule, T cell immunoglobulin and mucin domain‐3 (Tim‐3) inhibits mRNA and protein expression of HK2 in RAW264.7 macrophages via the STAT1 pathway, thereby inhibiting the mRNA level of IL‐1β and protein level of pro‐IL‐1β. When knocked down the HK2 expression using HK2 siRNA, the inhibition is reversed.^69^ Synthesis of novel andrographolide Beckmann rearrangement derivatives can inhibit the activity of HK2, thereby reducing the release of IL‐1β in RAW264.7 cells induced by LPS.^51^ In summary, HK contributes to NLRP3 inflammasome priming and activation; the agents that inhibit HK expression or dissociating from the mitochondrial membrane may prevent NLRP3 inflammasome‐mediated inflammation.

#### 3.1.3 PFK

PFK1 is one of the rate‐limiting enzymes in glycolysis, which can convert fructose 6‐phosphate into fructose 1,6‐phosphate.^70^ PFK1 has three isoforms: platelet (PFKP), muscle (PFKM), and liver (PFKL).^70^ PFKM is predominantly expressed in normal muscle and neuronal tissues.^71^ The long 3'‐UTR on the mRNA of PFKM can bind to microRNA‐21 and restrict the expression of PFKM, thus affecting the secretion of IL‐1β in BMDMs stimulated by *Mycobacterium tuberculosis* (Mtb).^72^ PFKFB3 synthesizes fructose 2, 6‐bisphosphate, which act as a powerful allosteric activator of PFK1.^73^ Transcription of PFKFB3 is enhanced by binding of the PFKFB3 promoter to Zinc fingers and homeoboxes (Zhx2), thereby promoting the release of IL‐1β in LPS‐stimulated BMDMs.^74^ Microglia are macrophages that reside in the central nervous system.^75^ Monocarboxylate transporters (MCTs) are also important glycolytic regulators that transport excess lactate out of cells. PFKFB3 can be promoted by MCT1 through Hif‐1α, thus promoting the secretion of IL‐1β by LPS‐stimulated primary and BV2 microglia. Overexpression of PFKFB3 rescued the siMCT1‐mediated reduction of IL‐1β expression and demonstrated the promoting effect of PFKFB3 on the release of IL‐1β from LPS‐stimulated microglia.^76^ In summary, the expression of PFK1 mainly promotes the maturation and secretion of inflammatory factors.

#### 3.1.4 PK

PK is the key enzyme in the last step of glycolysis, which catalyzes the reaction between phosphoenolpyruvate (PEP) and ADP to form pyruvate and ATP. PKM2 regulates the transcription of GLUT1, LDHA and other glycolysis‐related genes and ties to hypoxia inducible factor‐1α（HIF‐1α）to promote aerobic glycolysis in LPS‐treated macrophages.^77^ PKM2 induces the assembly of NLRP3 inflammasomes and release of inflammatory factors by phosphorylation of factor 2 alpha kinase 2 (EIF2AK2), which is well known to play a critical role in inflammasome activation.^47^, ^78^, ^79^ PKM2 knockdown or its inhibitor shikonin suppresses EIF2AK2 phosphorylation,^80^ thereby reducing casapase‐1 activity, inhibiting release of IL‐1β or IL‐18 in BMDMs stimulated by LPS and the NLRP3 inflammasome activator ATP.^47^ And interaction between NLRP3 and ASC (also termed as PYD and CARD domain containing PYCARD) is inhibited upon PKM2 knockdown in caspase‐1−/− BMDMs induced by LPS and ATP.^47^ It indicates that PKM2 regulates the production and release of proinflammatory factors in the NLRP3 inflammasome‐dependent pathway through phosphorylation of EIF2AK2. As the product of glycolysis, lactate contributes to the inflammatory process through multiple mechanisms.^51^, ^70^ For example, it activates NLRP3 inflammasome‐dependent inflammatory release, IL‐1β release and EIF2AK2 phosphorylation in BMDMs triggered by LPS.^47^ EIF2AK2 knockdown or addition of its inhibitor C16^81^ inhibits the phosphorylation of EIF2AK2 induced by lactate and the release of IL‐1β in LPS‐activated BMDMs.^47^ As the main bioactive component of Chinese wolfberry, *Lycium barbarum* polysaccharide (LBP) can reduce the expression of PKM2 protein in LPS‐induced RAW264.7 macrophages, but has no effect on the expression of PKM2 mRNA. LPS inhibited the ubiquitination of PKM2, possibly by downregulating the expression of ubiquitin ligases, including Nedd4L, Nedd4 and Gnb2. LBP interferes with the inhibition of PKM2 ubiquitination by upregulating the expression of Nedd4L, Nedd4, and Gnb2, thereby reducing IL‐1β production in LPS‐induced RAW264.7 macrophage.^52^ Deoxyelephantopin (DET), a naturally occurring sesquiterpene lactone from Elephantopus scaber, inhibited the nuclear localization of PKM2 and thus attenuated the release of IL‐1β from LPS‐stimulated RAW264.7 macrophages.^53^ IRD is a main isoflavone derived from the root of the plant Belamcanda chinensis (L.) Redouté, can target PKM2 and inhibit its downstream expression of p‐JAK1, p‐STAT1, p‐STAT3, p‐p65, iNOS and COX2, thereby inhibiting the release of IL‐1β from LPS‐treated RAW264.7 macrophages. PKM2 agonist DASA‐58 could abolish this inhibitory effect.^82^ In summary, inhibition of PKM2 expression mainly reduces the maturation and secretion of inflammatory factors.

#### 3.1.5 Lactate

Lactate is the end product of glycolysis and is derived from pyruvate by the enzyme lactate dehydrogenase (LDH).^54^ Lactate can induce phosphorylation of PKR,^47^ promoting protein expression of PKR, induces the NLRP3 inflammasome‐dependent IL‐1β secretion in nigericin, ATP, monosodium urate (MSU) crystals, or alum stimulated BMDM and THP‐1 cell.^24^ The protein levels of mature IL‐1β and active caspase‐1 were reduced by using 2‐DG or LDH inhibitor GSK2837808a, or by lactate dehydrogenase a (LDHA) specific small interfering RNA (siRNA). But the levels of NLRP3, ASC, pro‐caspase‐1 and pro‐IL‐1β did not change.^24^ However, in other studies, increased lactate did not promote the activation of NLRP3 inflammasome. In macrophages and monocytes, exogenous lactate reduced TLR4‐mediated induction of IL‐1β, NLRP3, and pro‐caspase‐1; activation of nuclear factor κβ (NF‐κB); release of IL‐1β; and cleavage of caspase‐1.^83^ Addition of lactate directly inhibits pro‐IL‐1β, NLRP3, and Caspase‐1 levels in LPS‐mediated human peripheral blood mononuclear cells, and also inhibited pro‐caspase‐1 cleavage, mature caspase‐1 proteolytic activity, as well as caspase1‐dependent process and extracellular release of IL‐1β. It is suggested that lactate has no promoting effect on the activation of NLRP3, and has moderate antagonistic effect. Other studies have found that exogenous lactate stimulation can inhibit the expression of PFKFB3 induced by LPS in BV2 cells, and also show inhibitory effect on the expression of IL‐1β.^76^ The results of these experiments are different, the possible difference is in the study of lactic acid, exogenous lactate may act as an exogenous inhibitor of NLRP3, and the increase of lactic acid caused by intervention methods has a promoting effect on the activation of NLRP3 inflammasome.

#### 3.1.6 HIF‐1Α

HIF‐1α is a decisive mediator of glycolysis, by inducting enzymes in glycolysis, especially HK2, PKM2, glucose‐6‐phosphate isomerase (GPI) and triosephosphate isomerase.^84^ HIF‐1α promotes priming of NLRP3 inflammasome by upregulating the expression of NLRP3,^85^ and is important for IL‐1β release. Overexpression of HIF‐1α and IL‐1β are found in THP‐1‐derived macrophages stimulated by palmitic acid; furthermore, knockdown of HIF‐1α can inhibit the proinflammatory effects of palmitic acid. NLRP3 inflammasome is activated and HIF‐1α overexpressed in RAW246.7 macrophages stimulated with LPS/ATP; GN44028, as HIF‐1α inhibitor, suppresses the expression level of HIF‐1α and NLRP3, thus IL‐1β release.^86^ Chaetocin, an antibiotic with epipolythiodioxopiperazines structure produced by Chaetomium sp,^87^ reduces the level of pro‐IL‐1β by inhibiting HIF‐1α, thereby affecting NLRP3 priming inflammasome and IL‐1β synthesis.^55^

GAPDH and ENO affects glycolytic flux, including glycolytic capacity and glycolytic reserve, which has been considered to involve in the full activation of NLRP3 inflammasome.^39^, ^41^ GB111‐NH2,^88^, ^89^ as a peptide‐based compound that inhibits both GAPDH and α‐enolase,^20^ restores metabolism downstream of glycolytic disruption, which is sufficient to suppress the inflammasome response by reinstating NADH generation and reducing mitochondrial ROS generation.^20^ Furthermore, KA and EB, as inhibitors of GAPDH and α‐enolase respectively, also affect glycolytic flux, and then the activation of NLRP3 inflammasome.^20^ However, inhibition activity of GAPDH or other enzyme in lower glycolysis could disrupt glycolytic flux and induce the activation of the NLRP3 inflammasome in primed murine bone marrow–derived macrophages.^20^ Taken together, the regulatory roles of glycolytic flux in NLRP3 inflammasome activation are still confused.

### 3.2 NLRP3 inflammasome activation promotes regulates glycolysis in macrophages

Key enzymes of glycolysis and end product (lactate) from the glycolytic pathway, promote NLRP3 inflammasome activation as mentioned above. Likewise, evidence shows that activation of NLRP3 also regulates glycolysis via several mechanisms.^15^

As one of the HK isoforms, HK1 expression is induced by NLRP3 inflammasome activator ATP and LPS but not with the AIM2 inflammasome activator poly (dA:dT) in BMDM. It indicates the specificity of NLRP3 inflammasome in activating HK1.^45^ Caspase‐1 cleavage assay in vitro combined with the proteomic analysis of caspase‐1 reveals those glycolytic enzymes including aldolase, triose‐phosphate isomerase, GAPDH, α‐enolase and PK are caspase‐1 substrates.^19^ Expression of GAPDH, aldolase, enolase and TIM was suppressed in peritoneal macrophages from wild‐type mice infected with *Salmonella typhimurium*, but not in macrophages from mice lacking caspase‐1.^90^ The targeting of caspase‐1 to glycolytic enzymes was also demonstrated in the diaphragm of mice with LPS‐induced septic shock.^19^ However, these results await further animal experimental confirmation. PFKFB3 synthesizes fructose 2, 6‐bisphosphate, which act as a powerful allosteric activator of PFK1,^73^ thus drive glycolysis.^15^ In macrophages, LPS and Aβ increased the rate of glycolysis and PFKFB3 expression, and these effects were counteracted by MCC950, a selective NLRP3 inhibitor.^91^ IL‐1β induced glycolysis activation by activation of PFKFB3 mimicking the role of LPS plus Aβ, which added the evidence to support the impact of inflammation on metabolomic profiles. This view was further supported by other observations that PFKFB3 and changes in glycolysis stimulated by LPS + Aβ were attenuated in BMDMs from NLRP3−/− and interleukin‐1‐receptor type 1‐homozygous knockout (IL‐1R1−/−) mice.^91^ In line with this notion, 3PO, the PFKFB3 inhibitor, attenuates glycolysis induced by LPS + Aβ. In summary, the NLRP3 inflammasome could modulate glycolysis by upregulating PFKFB3 expression in an IL‐1β‐dependent manner in macrophages.^15^

**Figure 2:**
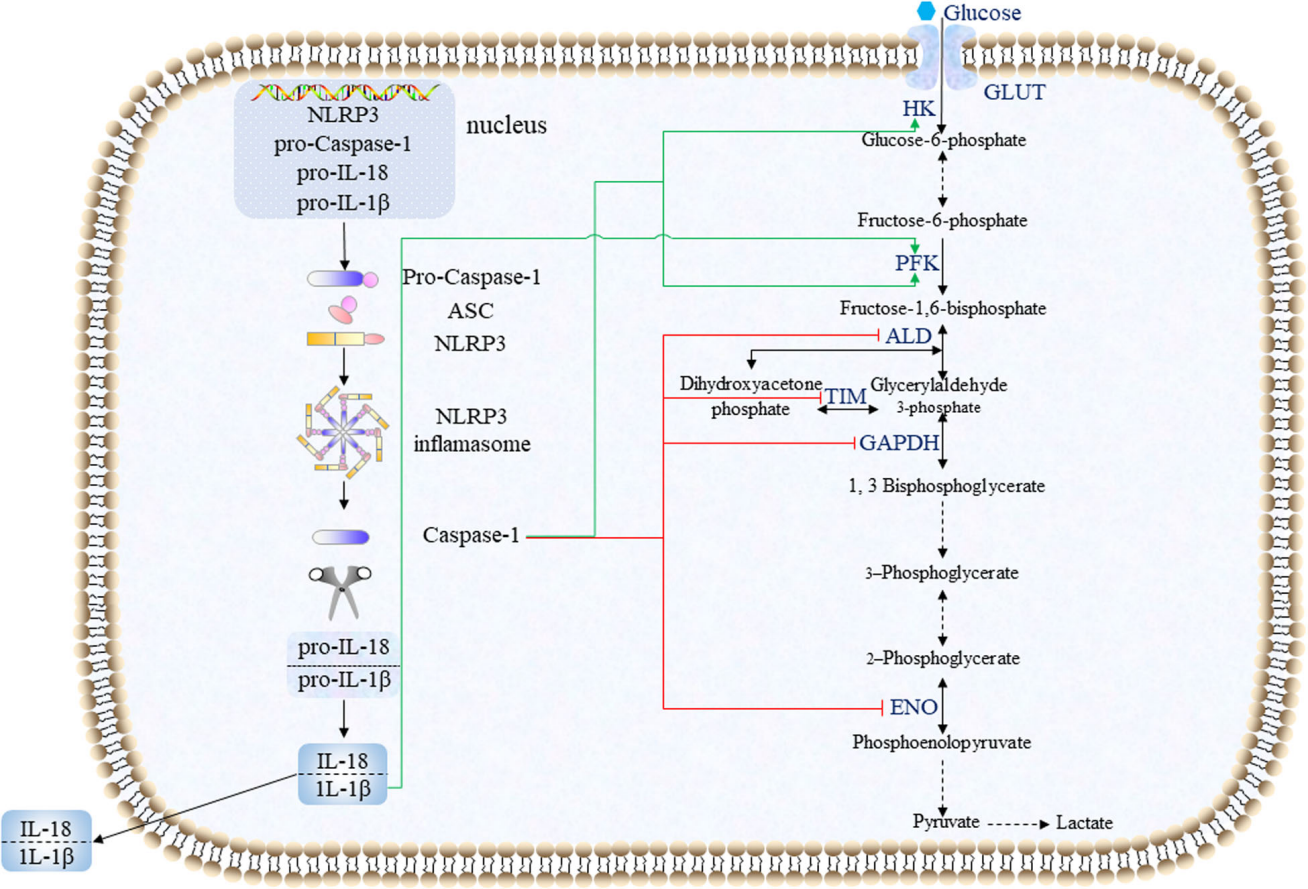
Schematic representation of the possible mechanism of NLRP3 inflammasome regulates glycolysis in macrophages. Caspase‐1 can promote the expression of key glycolytic enzymes HK and PFK and inhibit the expression of ALD, TIM, GAPDH and ENO. IL‐1β can promote the activity of PFK. ALD, aldolase; ASC, apoptosis‐associated speck‐like protein; ENO, enolase; GAPDH, glyceraldehyde‐3‐phosphate dehydrogenase; GLUT, glucose transporter; HK, hexokinase; IL‐18, interleukine‐18; IL‐1β, interleukine‐1β; NLRP3, Nod‐like receptor protein 3; PFK, phosphofructokinase; TIM, Triosephosphate isomerase

Research on the regulation of NLRP3 by glycolysis was introduced in‐depth above (Figure 2), but the detailed mechanism how NLRP3 regulates glycolysis is still to be explored. Additionally, activation of pro‐Caspase‐1 induced by cigarette smoke extract results in decrease in basal glycolytic flux and damaged glycolytic burst after LPS stimulation in vitro,^92^ which implies that the regulatory roles of NLRP3 inflammasome activation in glycolytic flux are controversial.

## 4 NLRP3 INFLAMMASOME–GLYCOLYSIS INTERACTION AFFECTS DISEASE THERAPY

NLRP3 inflammasome plays an important role in the occurrence, development and prognosis of many chronic inflammatory diseases, such as atherosclerosis, obesity, diabetes, Tuberculosis, rheumatoid arthritis (RA). Because of the interaction between NLRP3 inflammasome and glycolysis, regulation of glycolysis mediated NLRP3 inflammasome activation is a new way to intervene in these diseases.

### 4.1 Atherosclerosis

Macrophages are involved in the whole process of atherosclerosis.^93^ Activation of NLRP3 inflammasome promotes atherosclerosis.^93^ Atherosclerotic plaques are often hypoxic areas, accompanied by infiltration of inflammatory cells such as macrophages, which leads to the accumulation of hypoxic environment and hypoxia‐inducible factor in inflammatory areas.^94^ Hypoxia reduces autophagic degradation of pro‐IL‐1β, thereby stabilizing pro‐IL‐1β protein in LPS‐stimulated human macrophages. Hypoxia also enhances LPS‐ and cholesterol crystal‐induced IL‐1β secretion in human macrophages, and the increase can be limited by caspase‐1 inhibitor Z‐YVAD‐FMK, suggesting that caspase‐1 is involved in this process.^94^ Furthermore, the protein levels of HIF‐1α and HK2 were significantly elevated in macrophage‐rich regions of human plaques characterized by hypoxia, and levels of cleaved‐caspase‐1 and IL‐1β were also significantly elevated in this region. This suggests that hypoxia upregulates glycolysis and thus promotes the activation of NLRP3 inflammasomes in the plaque region.^94^ Oxidized low‐density lipoprotein (Ox‐LDL) promotes atherosclerosis by inducing macrophage foam cell formation and sterile inflammation.^95^ BMDMs and mouse peritoneal macrophages under Ox‐LDL treatment induce PKM2 phosphorylation and promote its nuclear localization. PKM2 shRNA or shikonin abolished Ox‐LDL‐induced mRNA expression of HIF‐1α target genes LDH, GLUT1, IL‐1β, lactate and secretory IL‐1β production, suggesting that PKM2 regulates aerobic glycolysis and inflammation. In THP‐1 cells and BMDMs, when TLR stimulation and inflammasomes are activated by ATP, liver X receptor (LXR) induces HIF‐1α at the mRNA and protein level. The upregulation of HIF‐1α not only affects the mRNA expression of GLUT1 and HK2, but also promotes the mRNA expression of IL‐1β and the secretion of mature IL‐1β.^96^ In monocytes and macrophages from patients with atherosclerotic coronary artery disease (CAD), increased glucose uptake and glycolytic flux promote the production of mitochondrial reactive oxygen species, which in turn promote dimerization and nuclear translocation of the glycolytic enzyme PKM2. PKM2 phosphorylates the transcription factor STAT3, thereby promoting the production of IL‐1β. The use of 2‐DG to inhibit glycolysis, scavenge superoxide or force PKM2 tetramerization can correct the pro‐inflammatory phenotype of CAD macrophages and reduce the production of IL‐1β.^97^ Endogenous oxidized phospholipids promote both OXPHOS and aerobic glycolysis in LPS‐stimulated macrophages and in Western diet LDLR−/− or APOE−/− mice, and promote mRNA overproduction of IL‐1β without ascertainment of apoptosis.^98^

### 4.2 Obesity

In cases of obesity, adipose tissue macrophages (ATM) change from the anti‐inflammatory M2 phenotype to the proinflammatory M1 phenotype.^99^ ATM also exhibits metabolic reprogramming characterized by elevated glycolysis and oxidative phosphorylation. Compared with lean mice, ATM from obese mice showed increased ECAR and glycolytic capacity, increased mRNA expression of HIF‐1α and key glycolytic enzyme GLUT1, and increased gene level of IL‐1β. Exposure of BMDM macrophages to saturated fatty acid palmitate increases glycolysis and HIF‐1α expression, ultimately leading to mRNA induction of IL‐1β, whereas 2‐DG inhibits this induction. Macrophage‐derived HIF‐1α plays a critical role in regulating ATM accumulation and local and systemic IL‐1β production in mice with macrophage‐specific HIF‐1α targeted deletion.^100^

### 4.3 Diabetes

Diabetes mellitus (DM) is an important and independent risk factor for the development of coronary heart disease (CHD), and the mortality of CHD is higher in patients with diabetes than subjects without diabetes2.^101^ Increased glycolysis due to hyperinsulinemia in patients with diabetes and insulin resistance.^102^ During diabetes, macrophages and other innate immune cells have a proinflammatory phenotype and are the underlying factors of various diabetic complications.^103^ Hyperglycemia also leads to the activation of NLRP3 inflammasome.^104^ In THP‐1 cells, PKM2 activators TEPP‐46 and glycolysis inhibitor 2‐DG could reverse the protein increase of NLRP3, IL‐18 and IL1‐β induced by hyperglycemia.^105^ The Glycolytic capacity and glycolytic reserve of peritoneal macrophages in diabetic mice and BMDMs with long‐term high glucose treatment tended to decrease, and BMDMs with high glucose stimulation could increase the gene expression of Toll‐like receptor 4 (TLR‐4), the gene expression and release of IL‐1β, and enhance the proinflammatory response of macrophages. But decreased the phagocytosis and bactericidal activity of macrophages.^106^ As previously describe, CORM‐3 inhibits activation of NLRP3 inflammasome by reducing activation of mTORC1, inhibiting glycolysis in BMDMs. CORM‐3 also reduced the elevated serum IL‐1β levels in streptozotocin (STZ)‐induced diabetic mice.

### 4.4 Tuberculosis

Mtb, the bacteria that causes tuberculosis (TB), is phagocytosed by resident alveolar macrophages (AM), and infiltrating monocyte‐derived macrophages (MDM) which then upregulate bactericidal effector functions.^107^ The ability of AM to switch to aerobic glycolysis is impaired in smokers infected with Mtb. Cigarette smoke extract treated human monocyte‐derived MDM also showed reduced metabolic activity and reserves, as well as an impaired glycolytic response to infection. The production of IL‐1β driven by glycolysis is reduced, and the antibacterial ability of Mtb is weakened.^108^ Suberanilohydroxamic acid, an FDA‐approved histone deacetylase inhibitor (HDACi), rapidly converted Mtb infected MDM cells to glycolysis in a short time, and increased the level of IL‐1β in the supernatants of Mtb infected MDM cells and AM cells from tuberculosis patients.^107^ Rifampicin and isoniazid are the first‐line treatment for tuberculosis.^109^ The emergence of multidrug resistant (MDR) Mtb forces treatment with toxic second‐line drugs.^110^ MDR‐TB can inhibit the conversion of macrophages to glycolysis by downregulating the mRNA levels of LDH, PFKFB3 and Aldoa in macrophages differentiated from mouse bone marrow cells, thus inhibiting the cell supernatant levels of IL‐1β associated with NLRP3 inflammasomes.^111^ Infection of macrophages by Mtb induces a transition to glycolysis, characterized by an increase in lactic acid content and an increase in the ratio of glycolysis to oxidative phosphorylation. The inhibition of glycolysis leads to the decrease of IL‐1β mRNA level in macrophages infected with Mtb, and also reduces the killing ability of macrophages to bacteria. Blocking or deletion of IL‐1R counteracts the effect of aerobic glycolysis on intracellular bacterial survival, suggesting that infection‐induced glycolysis limits the survival of M. tuberculosis in macrophages by inducing IL‐1β.^112^ Other studies have shown that Mtb can limit macrophage glycolysis and IL‐1β secretion by restricting PFK‐m through microRNA‐21, which contributes to its own invasion.^72^

### 4.5 Rheumatoid arthritis (RA)

RA is an autoimmune disease,^113^ macrophages are an important factor in RA.^114^ The expression of HK1 and HK2 mRNA of LPS‐treated THP‐1 was significantly inhibited by simultaneous silencing of HK1 and HK2 or by using Lenalidomide (LND), an inhibitor of HK, thereby inhibiting the release of IL‐1β. LND can also alleviate the CIA clinical signs of arthritis model DBA/1J mice, indicating that the regulation of glycolysis for anti‐inflammatory treatment of RA is a new idea.^115^ Cinnamaldehyde (CA) is a major component of cinnamon,^116^ which has anti‐inflammatory effect. CA inhibits the activity of HIF‐1α by inhibiting the accumulation of succinate in the cytoplasm by inhibiting the expression of the succinate receptor GPR91. Inhibition of HIF‐1α activity inhibited NLRP3 inflammasome assembly and IL‐1β production, attenuated inflammation in activated macrophages (Raw246.7) and synovial inflammation in adjuvant arthritis rats (AA).^117^ PKM2 was overexpressed at mRNA and protein levels in ED1‐positive macrophages in spleen and synovial tissues from arthritic rat. In classically activated rat and mouse macrophages, silencing Pkm2 by RNA interference resulted in increased phosphorylation of Stat1. Increased phosphorylation of STAT1 inhibits caspase‐1‐dependent IL‐1β maturation.^118^ Treatment of Dark Agouti rats with shikonin, a PKM2 enzyme inhibitor, or with RNA interference plasmids for PKM2 also improved arthritis in the dark Agouti rats.^119^

### 4.6 Conclusions

There is a complex interaction between metabolic reprogramming and immunity,^43^ making the current targeting of immune metabolism as an anti‐inflammatory strategy.^120^ Glycolysis and NLRP3 inflammasome activation regulate each other, which in turn are related to macrophage inflammatory, although their interactions are presently controversial. Therefore, inhibiting NLRP3 activation or glycolysis suppresses inflammation. Although it can be determined that glycolysis does contribute to NLRP3 inflammasome activation, the idea of regulating inflammasomes by glycolysis has been experimentally demonstrated in the treatment of many inflammatory diseases, such as atherosclerosis, obesity, diabetes, tuberculosis and Rheumatoid arthritis. Regulation of NLRP3 inflammasome by glycolysis may be a new idea for the treatment of chronic inflammatory diseases, and glycolysis inhibitors may also provide more options for the treatment of chronic inflammatory diseases. However, there are still some problems. For example, what the most critical substrate or enzyme of glycolysis in regulating inflammasome activity is and how NLRP3 inflammasome act on cellular metabolism are both still to be further elucidated. The exact target on which small molecule compounds bind during glycolysis to inhibit NLRP3 inflammasome activation remains to be further in‐depth study.

## CONFLICT OF INTERESTS

The authors declare that there are no conflict of interests.

## AUTHOR CONTRIBUTIONS

Qun Yu and Maojuan Guo drafted the manuscript. Xijuan Jiang and Bin Yu designed and supervise manuscript. Wenyun Zeng verified the contents and revised the manuscript. Miao Zeng, Xiaolu Zhang, Yue Zhang, Wenlan Zhang critically revised the manuscript. All authors reviewed and approved the final manuscript.
